# Contemporary issues in north–south health research partnerships: perspectives of health research stakeholders in Zambia

**DOI:** 10.1186/s12961-018-0409-7

**Published:** 2019-01-15

**Authors:** Tulani Francis L. Matenga, Joseph Mumba Zulu, J. Hope Corbin, Oliver Mweemba

**Affiliations:** 1 Department of Health Promotion and Education, University of Zambia, School of Public Health, P O Box 50110, Lusaka, Zambia; 2 Department of Health and Community Studies, Western Washington University, Miller Hall, 317B, MS 9091, Bellingham, WA 98225 United States of America

**Keywords:** Partnership, north–south, health research, power imbalances, achievements, challenges, trust

## Abstract

**Background:**

The late 1990s and early 2000s have seen a growth in north–south health research partnerships resulting from scientific developments such as those in genetic studies and development of statistical techniques and technological requirements for the analysis of large datasets. Despite these efforts, there is inadequate information representing the voice of African researchers as stakeholders experiencing partnership arrangements, particularly in Zambia. Furthermore, very little attention has been paid to capturing the practice of guidelines within partnerships. In this paper, we present achievements and highlight challenges faced by southern partners in north–south health research partnerships.

**Methods:**

A qualitative inquiry was employed using in-depth interviews developed using the Bergen Model of Collaborative Functioning with 20 key informants in Lusaka district in Zambia purposively sampled from a wide range of health research partnerships.

**Results:**

Partnerships produce benefits for southern partners, including evidence generation to influence policy, improved service delivery, infrastructure development and designing interventions to improve the healthcare of populations in greatest need. Most importantly, through partnerships, there is availability of financial resources to accomplish partnership goals. For success to be achieved, there must be effective communication and leadership, values and accountability that go into the process of partnership functioning. Trust interacts with different elements that create partnerships where there is co-ownership of study rewards. Challenging aspects of the interaction are largely due to funding mechanisms where 90% of the funding for health research is from northern partners. This funding mechanism results in power imbalances that lead to publication challenges, dictation of research agenda and ownership of samples and data leading to a general lack of motivation to collaborate.

**Conclusion:**

Mistrust has implications on joint working such that partners find it difficult to work together and produce results greater than their individual efforts. Property rights and resource sharing must be resolved early in the partnership and each partner’s contributions recognised. These findings highlight areas that partnerships need to focus on to make the most of guidelines on research partnership with developing countries.

## Introduction

The late 1990s and early 2000s have seen a growth in north–south health research partnerships, resulting from scientific developments such as those in genetic studies and development of statistical techniques and technological requirements for the analysis of large datasets [1]. This has been largely due to the inability and reluctance of African governments to fund scientific research and healthcare [2], which has resulted in major funding initiatives from northern governments and institutions for research on diseases such as HIV/AIDS, malaria and other neglected diseases responding to global academic interests and local health needs [3].

Partnership brings together multiple stakeholders based on common goals and shared intentions to produce an effect greater than the sum of their individual effects [4]. Health research partnership is an essential tool for improving healthcare in low- and middle-income countries (LMICs) and has the potential to play a significant role in addressing global health inequalities [5, 6]. Health research partnerships produce evidence that delivers population health changes that respond to critical needs and contribute to sustainable development outcomes in the world’s poorest countries [7]. Sustainable Development Goal 17, namely to “*strengthen the means of implementation and the global partnerships development*” [8], recognises multi-stakeholder partnerships as important vehicles for mobilising and sharing knowledge, expertise, technologies and financial resources to support the achievement of the sustainable development goals in all countries, particularly developing countries.

While many researchers acknowledge the worthy goals and benefits of international health research partnerships, they have also pointed out its practical challenges and limitations [9]. These challenges include power inequities, communication barriers, diverging research priorities, as well as a lack of capacity-building for southern partners [1, 3, 10, 11]. Centres dedicated to global health research partnerships with universities, hospitals and medical schools in LMICs have been established [3]. Despite these efforts, evidence suggests that partnerships face considerable obstacles in achieving the goals of equitable partnership as a result of power imbalances between northern and southern partners [2, 3, 10, 12].

Scholars describe these challenges in different ways, placing emphasis on the power of the north over the south, using different concepts to express this power discrepancy, e.g. the new imperialism – the north’s new way of extending its power [13] and unbalanced power relations [14, 15]. Crane [3] takes a step further by calling north–south partnerships a recolonisation of the south which creates intellectual dependency. This has been accompanied by growing debates on the ethics of conducting health research amid challenges of equity and concerns of post-colonial science in Africa [11, 16].

Literature on north–south partnerships and on ethics in international health research describes complex historical, political and economic partnerships between researchers from LMICs and high-income countries [1]. This research documents issues involving lack of informed consent, questionable social value and benefit sharing, power and equity differentials, poor community engagement, and limited access to data and export of biological samples [1, 3, 9]. These power dynamics have the potential to exploit research participants and African researchers [17] as they tend to favour collaborators in the north in terms of publication, authorship, capacity-building, data/sample ownership, roles and responsibilities [18, 19]. Meanwhile, research indicates that southern partners end up as data and sample collectors [2, 3]. Such cases may result in the reduction of the southern partner’s motivation to participate [20].

The majority of this literature is from stakeholders in northern countries and tends to focus on operationalising international guidelines and principles developed in an attempt to characterise good research practice in north–south health research partnerships [1]. These include the RAWOO Principles [21], the Canadian Coalition for Global Health Research [22], the Swiss Commission for Research Partnership with Developing Countries [23], the COHRED Research Fairness Initiative [24], and the Council for International Organisations of Medical Sciences (CIOMS) Ethical Guidelines [25]. These guidelines have increased amid calls for conducting ethically sound research in developing countries. Despite these efforts, there is inadequate information representing the voice of African researchers as stakeholders experiencing partnership arrangements [1, 26, 27], particularly in Zambia. Furthermore, very little attention has been paid to capturing the practice of these guidelines within partnerships. To address this gap, we conducted a qualitative research study with stakeholders involved in international health research partnerships in Zambia’s Lusaka district using a systems model, the Bergen Model of Collaborative Functioning (BMCF), as a framework for framing research questions and analysing the data. This paper aims to present achievements and highlight challenges faced by southern partners in north–south health research partnerships. In discussing the achievements and challenges, we utilise the Swiss Commission KFPE Guide for Transboundary Research Partnerships [23].

### Zambia’s health research system

Zambia’s health research system has undergone a great deal of transformation. In the past, there was no single governing structure that provided leadership in national health research. Currently, the National Health Research Authority, established under the Health Research Act No. 2 of 2013, is mandated to provide a regulatory framework for the development, regulation, financing and coordination of health research to ensure the development of consistent health research standards and guidelines for ethically sound health research in Zambia. Its functions include research promotion, research regulation, research coordination, research capacity-building, and research dissemination and knowledge translation [28]. The Zambia Forum for Health Research (ZAMFOHR), a non-governmental organisation launched in 2005, is another attempt at improving health research in Zambia. ZAMFOHR has had particular value in bringing researchers, research users, and research and health-equity institutions together to engage in research issues with government [29].

## Methods

We adopted a qualitative research approach using face-to-face interviews to explore factors that promote achievements and contribute to challenges in north–south health research partnerships in Zambia. Interviews were conducted with various stakeholders implementing health research activities in different parts of the country.

### Participants and recruitment

The study population included participants from various collaborations implementing health research activities related to HIV/AIDS, neglected tropical diseases, hepatitis, reproductive and sexual health, HIV prevention and maternal health. Participants included principal investigators, project coordinators/managers, laboratory managers, clinical researchers, and academic researchers and regulators from the Ministry of Health and the University of Zambia Biomedical Research Ethics Committee. Participants were at different career stages, with 2 researchers having been involved in health research for 3 years and 18 of the researchers having been involved in health research for more than 10 years. Participants from academia and health institutions had multiple roles such that, in addition to being part of health research partnerships, some were responsible for teaching, clinical work and management roles, while those from non-governmental organisations held specific roles such as project managers, laboratory managers and study principle investigators. Despite the participants being located in the capital Lusaka, research activities were conducted in different parts of the country with different institutions.

A purposive sampling strategy was employed, which involved selecting participants based on their expertise [30]. Using purposive sampling enabled the researcher to select health research stakeholders who played a significant role in at least one or several large international health research partnerships. In doing so, a sample which is known to be information-rich was selected to adequately inform the study. Sampling started by going through the ZAMFOHR online database to become familiar with researchers, institutions and projects/collaborations. Respondents for the interviews were then selected in consultation with the assistant dean’s office, University of Zambia, School of Public Health, and the co-authors OM and JMZ based on the inclusion criteria. Researchers were excluded if they had been involved in north–south health research studies operational for less than 1 year at the time of data collection, and where research studies had been completed more than 10 years prior to the commencement of the data collection.

### Data collection method

Primary data collection was through in-depth interviews with participants in Lusaka, over a period of 4 months between October 2017 and January 2018. A total of 20 interviews were conducted by the first author. A topic guide developed using the BMCF, which has been employed in similar projects [4, 10, 12, 31], was used to steer the interviews. The interviews covered a wide range of topics from the BMCF and some that emerged during the interviews. The themes explored included personal research career and experience of the collaboration, the mission of the collaboration, leadership of the collaboration, partner’s resource contribution, partner’s roles, responsibilities, challenges and achievements experienced in the collaborations, and factors of particular importance in collaborations between southern and northern partners. Follow-up questions were also used to get further clarification where necessary.

### Data analysis

All interviews were recorded digitally and later transcribed verbatim by the first author. The interviews were 30 to 90 min long. Transcripts and audio recordings were shared with co-authors for review and verification. The use of multiple researchers to validate results was important for checking mistakes [32]. Analysis was conducted mainly by TM and was supported by the co-authors through an interactive process including cross-checking and discussions. Analysis was conducted simultaneously with data collection, with initial analysis of early interviews informing the themes explored in those that followed. We followed a thematic analysis approach, which is a method for identifying, analysing and reporting patterns within data. This minimally organises and describes a dataset in detail and goes further to interpret various aspects of the research topic [33].

Transcripts were read multiple times for familiarisation and several meetings were held in Bergen, Norway, between the corresponding author and JHC, who has experience on partnership functioning. Particular attention was given to patterns and occurrences within the dataset. A codebook was also developed by the first author in agreement with the co-authors, based on the key questions and the theoretical underpinnings of the BMCF [4]. The coding process involved matching of codes with segments of data representative of the code carried out in Nvivo 12 data management software. The coded data was then collected into potential themes. The themes were then reviewed through checking if the themes were in relation to the coded extracts and the entire dataset before arriving at the final themes [32].

The revealed final themes and the results were written according to the framework and literature that made meaningful contributions to answering research objectives. The following organising themes were presented: mission of the partnership, financial resources, partner resources, partner roles and responsibilities, input interaction, and synergy and antagony.

### Ethics approval

This study was approved by the University of Zambia Biomedical Ethics Committee and the National Health Research Authority in Zambia. Signed informed consent was obtained from all participants before each interview and all personal details were removed to ensure confidentiality.

### Conceptual framework on partnership functioning

**Fig. 1:**
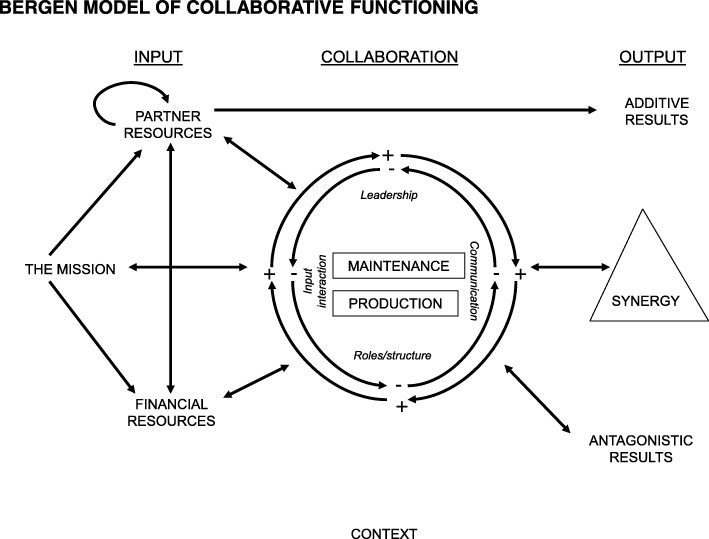
Bergen Model of Collaborative Functioning [12]

The BMCF provides an analytical frame for examining collaborative arrangements [4, 10, 12, 31]. The model depicts the inputs, throughputs and outputs of collaborative functioning as cyclical and interactive processes within the system (Fig. 1). The inputs to a partnership are its mission, partner resources and financial resources. Mission refers to the agreed-upon approach of the partnership to address a specific problem or issue. Partner resources refer to the skills, knowledge, power, commitment, connections and other attributes that human resources contribute to the partnership. Financial resources encompass all monetary and material investments in the partnership [12].

The throughput section is the collaborative context. Inputs enter this context and interact positively or negatively as they work on the maintenance (administrative tasks) and production (relating to the collaborative mission) activities of the partnership. The collaborative context is shaped by the interaction of four elements, namely the inputs themselves as they engage in work, the leadership, roles and procedures, and communication. These four elements can interact positively or negatively, creating dynamic and reinforcing cycles within the collaborative context [12].

The outputs of the collaborative context may be synergy and/or its opposite, antagony, in which the costs of partnership are perceived to outweigh the benefits [4]. The term ‘synergy’ is often employed to describe the multiplicative interaction of people and resources to solve problems that cannot be tackled by any of the partners working alone, which adds to the partnership [4, 10, 12]. Antagony is not the mere failure to produce synergy, it is the wasting of partner and financial resources to the extent that more is consumed in the process of collaborating than is produced, it subtracts from the partnerships [4, 10, 12].

The BMCF has previously been used as an analytical frame to examine case studies of several collaborative working arrangements, namely in Tanzania to assess an HIV/AIDS organisation’s history of north–south partnership [12], in Kenya in the implementation of the Community-based Health Management Information Strategy project [34], and in Botswana to explore achievements and challenges of the partnership on a safe circumcision programme establishing how the mission and functioning of the partnership contributed to the actual outcome [10]. In these studies, learning about both synergy and antagony created an opportunity for partners to reflect on what went wrong and what could have been done differently.

In particular, the study focused on contribution to the collaboration (input), how contributions interact to maintain activities of the collaboration (throughput) and the outcome of the collaboration (output). The study explored some of the elements at each stage of the partnership, i.e. input, throughput and output, that are responsible for creating synergies for one partner while creating antagony for the other. The model provided the basis for framing the research objective and questions of the study as well as the interview guide. By implication, the data was analysed deductively (based on the main categories of the model) while at the same time inductively deriving themes from the data.

## Results

The revealed themes were written in relation to the global and organising themes in the BMCF and in consultation with literature on health research partnerships that made significant contributions to the research objective. The results presented show how input, throughput and output processes interact with each other in producing both achievements and challenges as summarised in Table 1.

**Table 1:** Themes organised according to the Bergen Model of Collaborative Functioning and from interviews

Global theme	Organising themes	Themes from interviews
Input	Mission	Health research to improve healthcare
		Desire to authentically contribute to mission
		Personal livelihood
		Professional/career goals
	Partner resources	Southern partner’s capacity to contribute to the mission underutilised
		Responsibility of northern partners to build capacity
	Financial resources	Ninety percent funding from northern partners
Throughput	Input interaction: power	Power to delegate tasks
		Power to dictate timeline
		Power to dictate expenditures
		Communication: transparency
Output	Synergy	Infrastructure development
	Antagony	Authorship and publication of study results
		Access and use of health research data

### Input

#### Mission

##### Health research to improve healthcare

The motivation for health research partnerships is to improve the quality of healthcare for the most disadvantaged populations. Through health research, information is generated and used to influence policy and design interventions that directly benefit the community. These achievements are linked to the mission of the partnership. For example, partners were motivated to achieve the 90–90–90 target in the fight against HIV/AIDS; according to this mission, by 2020, 90% of people who are HIV infected will be diagnosed, 90% of people who are diagnosed will be on antiretroviral treatment and 90% of those who receive antiretrovirals will be virally suppressed:

“*We have the 90–90–90 target. So it’s a day-to-day thing of trying to come up with new ideas on how we are going to scale up on the viral loads.*” (IDI 07 Laboratory Manager)

Establishing of the mission together is important for the partnership because interventions are designed using funds from northern partners. Northern partners have resources which southern partners do not have to develop such health interventions. Working with their northern counterparts, they generate evidence with the intention to influence successful policy development and improve service delivery. This is achieved not only through a supply of financial resources, but also through ideas from multiple partners with different experiences:

“*The issue of partnering with people from the north is that there is a strength of funds. The assumption is that we will be able to test interventions and once we find that what we are testing is effective, it works, we hope that it can actually be taken up by relevant authorities. It can be translated into policy.*” (IDI 04 Academic Researcher/Scientific officer)

For partnerships to succeed, there is a need for both partners to be fully engaged, although southern partners understand the local systems better as they are closer to the communities experiencing health challenges. Northern partners, on the other hand, have experience working in similar settings that have implemented related projects. As a result, local researchers are better placed to lead the implementation on the ground, being aware of what is acceptable to the communities. Interviewees emphasised the importance of local inputs to ensure that the research is relevant to the communities they targeted:

“*It’s key to have strong local input because, at the end of the day, I think that usually, all parties want to improve local health and well-being but sometimes the external party may not know how to do this and might be a bit off in the approach or they might think that something is more priority than it is.*” (IDI 09 Principle Investigator)

##### Desire to authentically contribute to the mission

Health research partnerships have the potential to lead to long-lasting success where findings are translated into action and policy. Accomplishments such as providing the Ministry of Health with information to improve the health systems motivates southern partners. Interviewees indicated their desire to participate in partnership through contribution to scientific knowledge and thus improve their practice:

“*My motivation first started with my opportunity to do research courses such as epidemiology which sort of opened up my mind that as a practicing doctor I may not be enough; I need to find answers especially for common problems. So, I found that collaborating with my colleagues from the north was helping me meet my goals.*” (IDI 10 Clinical Researcher)

Despite achievements of international health research partnerships alluded to by all interviewees, many of them were of the view that health research between northern and southern partners was still flawed, with power inequality largely due to funding mechanisms:

“*It’s a very good thing to collaborate with international health researchers because they help in the transfer of knowledge but the challenge is that there are unequal power relationships.*” (IDI 08 Academic Research)

##### Personal livelihood

For many researchers in developing countries, research is a source of income and employs a number of people to carry out different aspects of the project. If there is a donor who is giving the money, they have the power in most cases and there is a lot of compromise by local researchers receiving the funds. This has often worked to the disadvantage of many southern partners who may not be able to speak about power inequality for fear of losing their source of income:

“*Research employs a lot of people, gives people a livelihood, and provides the lights. So we look at it as a source of revenue.*” (IDI 09 Principle Investigator)

##### Professional/career goals

In addition to having a wider purpose of the partnership, individuals have personal ambitions and goals. Many of the interviewees benefited at an individual level in terms of professional advancement, where a number of them have pursued higher education through masters and post-doctoral sponsorship. They were now able to publish in international journals and were often called upon by international partners to collaborate on other research projects. Such achievements warranted the need to stay in the partnerships:

“*I think we turn a blind eye to certain things and sometimes you pretend like you haven’t seen certain things. You may know that these people are undermining me, but I don’t know maybe it’s for the sake of being on that project and because you are hoping that by virtue of me being there at least, I will be able to publish.*” (IDI 04 Academic Researcher/Scientific officer)

Speaking about these power inequalities may lead to some individuals being excluded from the partnership, which most southern partners avoid:

“*Sometimes people are systematically excluded from the partnership depending on what individuals think. If they think you are controversial. Sometimes you can even be very constructive but if people think that you are asking too much, that can also lead to you being systematically excluded from the partnership.*” (IDI 03 Academic Researcher)

#### Partner resources

##### Southern partner’s capacity to contribute to the mission

All partners need to contribute to achieving success through funding, implementation, monitoring and building of the knowledge base. Northern partners dependent on southern partner’s local skills and knowledge of the context as they conduct research. This mutual dependency was mentioned by interviewees signifying their contribution in terms of local knowledge, network building and local expertise:

“*Money can be there but if the local experts that are going to implement it are not there, that means that money will not yield any results…. One brings the resources in terms of financial resources; we have the local resources to implement the activities as well as the skill.*” (IDI 15 Clinical Researcher)

Despite this mutual dependency being echoed, southern partners felt their skills were often underutilised based on the assumption that they lacked the needed expertise to contribute:

“*But there is also the aspect of people from the north also having that kind of superiority complex, they kind of feel they know it all…. Everybody has different skills, different strengths and so even the people from the south there is something very unique that they bring on the table.*” (IDI 04 Academic Research/Scientific Officer)

##### The responsibility of northern partners to build capacity

By working with northern partners, there is a flow of research skills especially to less experienced young researchers through mentorships programmes. Many interviewees emphasised the responsibility of northern partners to continue building capacity among southern partners as there was still a lack of expertise in developing countries.

“*Because this research is being done in Zambia, when they are coming, we expect them also not only to get just data from this country but also, they must build capacity as well among the locals. So really, to me, when research is coming to the county, in most cases we would want to see that there is a component of capacity-building, and one of them is through the involvement of local researchers.*” (IDI 05 Ethics Review Committee Member)

#### Financial resources

According to the BMCF, one of the key ingredients to a partnership is a broad range of participation from diverse partners and a balance of human and financial resources. Financial resources are the most important aspect respondents mentioned as being important for partnership:

“*We cannot run away from the fact that we need funds. For example, the reagents we use in the lab, we need to procure those things, you need to keep your staff going, they need to survive, infrastructure, all these things. You need power to be running, you need consumables as well, to keep going, all those come with a cost.* [Northern partner] *has been very good to look at that and ensure that everything is running.*” (IDI 07 Laboratory Manager)

##### Ninety percent funding from northern partners

Despite the availability of financial resources from northern partners, interviewees pointed out that a funding mechanism where 90% of the funding was from the northern partner was a challenge, which often led to power imbalances:

“*We do not fund research in this country; research is not a very big priority to our country. So most of the money that comes in is from our partners in the north. In addition, our partners sometimes they will say we have money and this money must be used on this and this kind of research. So that the local researchers have to adapt* [laugh] *to what the demands of the funders are.*” (IDI 05 Ethics Review Committee Member)

### Throughput

As the inputs interact during production and maintenance activities through time and roles, power struggles are also manifested. This is shaped by the interaction of roles, leadership and communication. There can be both positive and negative experiences as partners interact to work together.

#### Input interaction: power

##### Power to delegate tasks

Every type of partnership arrangement has particular structures and separation of roles and responsibilities between partners. Partner roles and responsibilities must be spelled out at the beginning of the partnership. However, these partner roles and responsibilities in some partnerships may sometimes cause challenges in terms of power imbalances where the southern partners remain relegated to the role of data collectors and required to send collected data to northern partners for further analysis:

“*I remember one of the conferences we went to outside Zambia. We had been there for 5 days, at the end of the day we were allocating duties, who does what. We were almost done then this professor from one African country just stood up and said, ‘Have you realised that all the donkey work has gone to Africa?’*” (IDI 03 Academic Researcher)

##### Power to dictate the timeline

Tension is often created at all stages of the partnerships, including at the start of the research. In setting up procedures, southern partners find themselves facing long ethical clearance processes. This in itself puts southern partners under pressure to meet the expectations of northern partners within agreed timelines. This results from a lack of consideration of the long procedures required to get clearance from institutional review boards:

“*Some of the major disappointments have been unrealistic expectations by the northern partners sometimes. I think they have to understand the environment in which we are working in and sometimes it may not be the fault of the project implementation group but just the bureaucracies around achieving what the project is meant to do.*” (IDI 14 Project Manager)

##### Power to dictate the expenditures

Lack of funding means that southern partners do not have the power to decide how finances are spent. Northern partners dictate how money meant for research should be spent and on what without the explicit involvement of the southern partner:

“*The principal investigator will be a northern partner and, us as Zambians, we are just co-investigators so that in itself sometimes has its limitations in the sense that, whereas we could be involved in the initial budgeting process, you may find that we have no control over the budget per say and in some cases you find that the money comes from the northern partner. So, our colleagues tend to have an upper hand.*” (IDI 10 Clinical Researcher)

### Output

Outputs of a partnership are the rewards that come with working together. The BMCF shows the two kinds of outputs in a partnership, i.e. synergy and antagony.

#### Synergy

##### Infrastructure development

Synergy is the most desired outcome for collaboration. By working together, partnerships have created more than they would achieve working in isolation. Achievements include new structures, such as laboratories, which were never there before and are now serving the wider community:

“*These laboratory facilities have enabled tests to be performed in the area of HIV/AIDS and thus improve service delivery to the community. They have been able to build a scientific lab which is still currently standing at the moment and this is a lab where you can do very high tech tests.*” (IDI 19 Clinical Researcher)

#### Antagony

##### Authorships and publication of study results

Fair distribution of authorship has been a concern in international health research partnerships between northern and southern partners where southern partners have been left out of authorship where they have significantly contributed. Authorship practices in international health research can be even more challenging given the variety of roles and responsibilities of researchers from LMICs and high-income countries.

“*The worst-case scenario is where some people write nothing completely, but they are part of the publication because they are part of the partnership. So usually young researchers like yourself you are told by the senior research people to say, ‘Do the work, after you have done the work you need to include everyone’. It’s more of like a political decision based on consortium or partnership arrangements.*” (IDI 03 Academic Researcher)

##### Access to and use of health research data

Ownership of data and biological samples has been another major discussion in health research partnerships and still continues to present challenges in partnerships. Although so much has improved with the coming of the National Health Research Authority in regulating health research in Zambia, some partnerships still experience lack of access to health research data once sent to partners for further analysis:

“*Once I get to do the quality control, I send that report to* [northern university] *…Once those recordings of those interviews go, we do not have access to them. The one who gets to decide what happens to the data after analysis is the* (northern)-*based principle investigator. Although it will be done in collaboration with the local principal investigator but the main manager of that and control is done by the* [northern] *partner.*” (IDI 11 Project Manager)

##### Communication: transparency

Effective and regular communication holds a partnership together. Without it, it is almost impossible to maintain effective partnership functioning. Transparency and communication are linked to leadership styles where more power is given to the northern partner who makes the major decisions for both partners even though both partners maybe equal applicants of the research grant. Leaders who are not transparent often cause tension in partnerships, which may demotivate partners:

“*In a way, these issues rotate around funding and leadership as well because funding, I will say definitely whoever funds calls the shots…. Leadership because when you have all those financing issues, you know there also leadership issues, because if there is strong and good leadership you shouldn’t have financial problems.*” (IDI 18 Academic Researcher)

The power imbalance described above creates a collaborative environment which does not nurture trust between partners. Interviews pointed out that trust is a pillar on which partnership is built upon and sustained, if the trust was broken through mismanagement of funds, especially by the southern partner, the partnership would often come to an end:

“*If at all they* [northern partners] *sense anything to say that the people we are going to be dealing with may not be handling the monies properly. They may not have the time to invest in the research, they very easily pull out.*” (IDI 19 Clinical Researcher)

## Discussion

This paper used BMCF as a framework for framing research questions and analysing the data to show how input, throughput and output processes interact with each other in producing both achievements and challenges. The Swiss Commission for Research Partnership with Developing Countries [23] suggests mechanisms for managing health research partnerships to maximise synergy through 11 principles (namely set the agenda together, interact with stakeholders, clarify responsibilities, account to beneficiaries, promote mutual learning, enhance capacities, share data and networks, disseminate results, pool profits and merits, apply results, and secure outcomes). We discuss the findings using some of these principles and present achievements and highlight challenges faced by southern partners in north–south health research partnerships.

The more ambitious the mission, the more important it is for all parties involved to achieve positive results from their work [23]. In the case of the 90–90–90 target in the fight against HIV/AIDS, the findings suggest that having a clear goal at the beginning of the partnerships helps partners commit to working together. This also serves as a motivating factor and a reason to continue partnering. In addition, working with northern partners mobilises the necessary resources for infrastructure development, knowledge generation for policy development and designing the health interventions needed to address local health needs [6, 26]. In this way, respondents felt that they were making a difference in the area of healthcare. Oldham [35] suggests that access by scientists in the south to knowledge and expertise in the north, with the intention of applying this knowledge to local challenges, provides a significant benefit to research partnerships.

As a result of partnership profits and merits, researchers in southern countries often seek out collaboration with researchers in northern countries to tap their expertise [26]. By way of engaging in research activities, southern partners get exposed to networks and processes of obtaining funding for new research, identified by Corbin et al. [12] and Katisi et al. [10] as the ability of synergy to generate more positive and greater interaction. Furthermore, with international health research partnerships comes a moral imperative to engage in efforts to translate evidence into policies and programmes that benefit populations [26]. When conducted properly, health research becomes a tool for development that benefits the community, especially in the developing world. Thus, equitable and well-governed research partnerships are an effective means through which to ensure that quality research results are translated into policy and have an impact on health disparities [22]. However, such profit distribution becomes challenging in cases where several of the parties involved lay claim to the same piece of the cake [23]. For example, respondents stated that they were active participants in all research activities, from collecting data to producing the first draft of the report, but left out in activities such as data analysis and publication. A situation that requires commitment to fair allocation of research benefits to all parties involved.

Any partnership ultimately depends on each partner contributing what they are particularly skilled in doing. This division of work makes it necessary to clarify and assign the responsibilities of partners [23]. Southern partners guide implementation of research activities on the ground while northern partners decide how financial resources are spent and which area of research partners go into. For southern partners, active participation in the partnership goes beyond data collection, it includes an overall contribution to the mission through programme implementation and monitoring as well as building the knowledge base. In addition to contributing to the larger mission, southern partners indicated their desire to contribute to the mission by doing tasks that utilised their skills. These partnership roles and responsibilities in some partnerships brings about challenges where southern partners are mostly delegated to lower tasks such as data collection while northern partners are mostly involved in the analysis of data and publication of the study results [36, 37]. One respondent compared this unfair assignment of roles and responsibilities to “*donkey work*”. Similarly, participants in Parker and Kingori’s study [1] expressed a comparable concern of southern partners being relegated the role of “*a glorified field worker*” responsible for collecting data but being excluded from the creative science. Although none of the respondents linked this unfair distribution of roles and responsibilities to post-colonial relations, the term ‘donkey work’ eludes clearly to unequal relations between partners in north–south health research partnerships. This unevenness in the allocation of tasks and responsibilities creates synergies for northern partners who receive recognition for their contribution and at the same time creates antagony for southern partners.

Capacity-building is a significant benefit of international health research partnerships, leading to strengthened capacity among individuals, institutions and systems [29, 38], and has been recognised as an essential part of working together [11, 35, 39]. Notably, organisations that promote partnerships through research funding programmes, such as Canada’s International Development Research Centre, helps to ensure that research occurs collaboratively and that resources are available to develop capacity in countries that have limited resources [40]. Through such partnership efforts, southern health researchers are able to improve their research skills and advance their careers. However, a major concern over the years has been that, despite increased investment in research programmes with multiple international partners, there is still less advancement in LMICs accruing their own research capacity and strengthened systems of health to protect their populations, as Ogundahunsi et al. [41] notes. A continued need for capacity-building for southern partners was emphasised, with many considering capacity-building as essential and its absence in collaborative arrangements viewed as undesirable.

The principle of promoting mutual leaning in view of capacity development can be even more challenging when trying to create a learning culture that complies with the different perceptions and cultural backgrounds of partners involved [23]. Muldoon [11] argues that the assumption implied in many collaborations that capacity needs to be built in the south while northern researchers are always ‘perfectly qualified’ does not hold. It undermines the opportunity for change when northern personnel, as ‘capacity providers’, are unable to admit to need, and southern researchers, as ‘receivers’, are not acknowledged for existing capacity. The situation is further exacerbated if the message is that southern need is caused by inferiority of abilities rather than simply a skills or technology deficit. Noticeably, some respondents reported that their capacity was often built on the assumption that they do not know and northern partners are superior to their counterparts, thus creating a paternalistic kind of capacity-building which creates a north–south dependency [6, 11]. This partnership model mirrors a post-colonial relationship based on old traditions of northern superiority over southern partners [11], as the ‘little brother effect’ [42] and as ‘Cinderella and her stepsister’ [20]. Such concerns do not fall short of support from the Swiss Commission [23], indicating that the days when research partnerships were understood as vehicles for a one-way transfer of knowledge and technology from north to south are over. The focus should now be on increasing both knowledge and know-how, while at the same time developing the capacities of all parties involved, including all stakeholders and junior scientists.

The principle of accounting to beneficiaries is still challenging in view of the assumption that the one who takes has to account to the one who gives. This upward accountability formula is neither suitable nor effective as it fails to take into account the fact that relevant research delivers benefits both to society and to science [23]. Reports show that, within partnerships, systems are oriented more towards ensuring accountability according to funders rather than adhering to collaboration theories [16]. Being accountable to a specific group of beneficiaries can trigger an important echo, leading to enhanced and genuine partnerships, new research questions, and to broader and deeper dissemination of results [23]. However, this one-way accountability can lead to mistrust between partners, where southern partners are held accountable to northern partners with regards the use of funds, while northern partners are not. This is a perspective reported by Walsh et al. [15], where donors did not trust southern researchers to manage funds and account for the research budget, instead placing more trust in northern partners. This practice does not nurture trust between partners and is linked to leadership styles where more power is given to northern partners who make major decisions for the partnership. While organisations such as ZAMFOHR are taking a leading role in health research in Zambia [29], leadership in many instances has remained in the hands of northern partners and reflects a real challenge in reality. This evidence suggests that more still needs to be done in building equitable and effective north–south health research partnerships in view of the Health Research Act of 2013 [43], which has called for local leadership in health research.

Jones and Barry [44] found trust to be essential in the production of synergy and recommends that trust-building practices be purposefully built into the functioning of the partnership at the beginning and maintained throughout its work. Its absence raises concerns regarding hidden agendas of partners which can hinder success. This can lead to lack of co-ownership of health research data and intellectual property rights. As northern partners take possession of health research data, ethical concerns arise around who has the right and authority to decide how data should be interpreted and shared [45, 46]. In Kenya, for example, a dispute, which eventually ended up in a court, involved a Kenyan researcher alleging fraud and theft of his research materials against eight Oxford University scientists. The stolen material consisted of children’s blood and tissue materials, which were allegedly taken from a Nairobi orphanage laboratory [45]. Respondents felt there was a greater need for effective and regular communication with clear memoranda of understanding at the beginning of the partnerships stipulating how data should be shared and who makes decisions regarding data sharing and dissemination. This builds a sense of mutual trust which enhances transparency in often unequal relationships and fosters the flow of information based on the principle of sharing data and networks [23].

Research priority-setting is a major challenge facing partnerships and has been echoed as often leading to inequitable and unethical partnership dynamics [26]. Common practice is a tendency for the partners with the most access to resources to set priorities based on their own interests, which might not reflect the actual priorities of the countries or communities in which the research is taking place [47]. Similarly, in some countries with weak health systems, foreign donors often set priorities without consulting local stakeholders [48]. Costello and Zumla [16] state that foreign domination in setting research priorities and project management may have negative consequences which outweigh the obvious benefits of research findings. The Ministry of Health, through its National Health Strategic Plan 2017–2021 [49], has set national health research priorities to guide governments and cooperating partners funding health research institutions, as well as researchers and other stakeholders, on the areas of research that would best respond to Zambia’s health needs; nevertheless, priority-setting has largely remained in the hands of the funder. Cases are rare where collaboration involving two research groups that contribute equally to funding have an equal scientific capacity and share the same interests. In such cases, asymmetry is inevitable and a fact, but its negative impact can be reduced by jointly determining research questions, approaches and methods [23].

At the root of these disparities obstructing the full utilisation of the Swiss principles is the power struggle experienced by southern partners due to funding mechanisms that have long dominated collaborative arrangements, where 90% of the funding comes from northern partners and sent directly to research institutions, usually without an explicit requirement that the research is aligned to national priorities [15]. This kind of funding mechanisms may lead to poor capacity-building and inaccessibility of results from samples/data that could facilitate research progress for most developing countries [14, 50]. Similarly, outdated practices around intellectual property and publication rights means that partnerships may have little benefit for less-resourced partners and the communities they represent. This study, like many others [12, 15, 26, 51], confirms that power imbalances and inequities exist at each stage of the research process – from funding to agenda-setting, data collection, analysis and research outputs – which outweigh the benefits of the partnership. This in itself may generate resentment and a sense of exploitation for southern partners [52].

## Limitations

One limitation of the study is that the study was conducted in Lusaka with a small sample of respondents. Therefore, the findings may not represent the experiences of researchers based outside this study setting. However, the results of the study may be used as a learning resource. Another limitation is that it focused on the experiences of southern partners only. The inclusion of northern partner’s experiences would have enabled the study to make a comparison of what partners thought about collaborations. However, there have been several studies that have included northern partner’s perspectives and have reached the same conclusion, which gives us confidence that findings are within the larger body of literature. Further, there are few perspectives from the community, who are the ultimate beneficiaries of health research. Research is therefore needed to include the perspectives of people or communities whom collaborative health research partnerships serve and how they are involved in the research process. Further research is also needed to find out if collaborations have any meaningful impact upon the people or communities they serve.

## Conclusion

The existence of challenges in health research partnerships has persisted over the years and co-occur with achievements benefiting one group more than the other. To improve relations in north–south health research partnerships there is a need for leadership styles that foster mutual trust. All actors need to contribute together to achieve success through programme implementation, funding, monitoring and building the knowledge base. From this study, we conclude that two factors have an impact on limiting the achievement of successful partnerships; firstly, lack of trust and transparency leads to ethical concerns around who has the right and authority to decide how data generated from health research studies should be interpreted and shared and how financial resources are spent. Secondly, power is likely to be associated with the ability of partnerships to actively engage diverse partners, to create an environment that fosters productive interactions between partners, and to facilitate meaningful participation in the partnership’s work. However, unequal power relations that often favour northern partners can limit the ability of partners to fully engage in activities that produce benefits.

Consideration of factors that may cause challenges in north–south health research partnerships aids in inspiring dialogue and reflection on issues that are rarely the focus in traditional evaluation methodologies. Doing so can further create a new form of partnerships based on trust and transparency led by effective leadership and communication. Further, such a move may also help strengthen national legislation in Zambia, such as the National Health Research Act of 2013, to address the structural inequities and power imbalances in health research partnerships. These findings also highlight areas that partnerships need to focus on to make the most of guidelines on research partnership with developing countries.

## Abbreviations

BMCF: Bergen Model of Collaborative Functioning
LMICs: low- and middle-income countries
ZAMFOHR: Zambia Forum for Health Research
